# Interoperability and Considerations for Standards-Based Exchange of Medical Images: HIMSS-SIIM Collaborative White Paper

**DOI:** 10.1007/s10278-019-00294-0

**Published:** 2019-11-25

**Authors:** Kenneth R. Persons, Jason Nagels, Chris Carr, David S. Mendelson, Henri “Rik” Primo, Bernd Fischer, Matthew Doyle

**Affiliations:** 1 Mayo Information Technology, Mayo Clinic, Rochester, MN USA; 2 Manager Clinical Program at HDIRS, Ontario, Canada; 3 Director of Informatics at RSNA, Chicago, IL USA; 4 Icahn School of Medicine at Mount Sinai, New York, NY USA; 5 Primo Medical Imaging Informatics, Inc., Chicago, IL USA; 6 ITH Icoserve Technology for Healthcare GmbH, Innsbruck, Austria; 7 Research and Development, Epic, Madison, WI USA

**Keywords:** Medical images, Interoperability, Image exchange, Standards-based image exchange, Standards-based interoperability, DICOM, FHIR, Governance, Canada Health Infoway, ELGA, RSNA Image Share, XDS, XDS-I, XCA, XDS repository, XDS registry, WIA, Web-based Image Access, VNA, Diagnostic image repository, Austrian Radiology Archive, Health information exchange, Personal health record

## Abstract

This white paper explores the considerations of standards-based interoperability of medical images between organizations, patients, and providers. In this paper, we will look at three different standards-based image exchange implementations that have been deployed to facilitate exchange of images between provider organizations. The paper will describe how each implementation uses applicable technology and standards; the image types that are included; and the governance policies that define participation, access, and trust. Limitations of the solution or non-standard approaches to solve challenges will also be identified. Much can be learned from successes elsewhere, and those learnings will point to recommendations of best practices to facilitate the adoption of image exchange.

## Introduction

This white paper explores the considerations of standards-based interoperability of medical images between organizations, patients, and providers.

Care is increasingly delivered across multiple organizations. Patients move between health systems for various reasons, including second opinions, delivery of specialized care, continuity of care, and unplanned transitions of care. While Vendor Neutral Archives (VNAs) [1] and medical imaging standards [2] have helped provide the infrastructure for managing and exchanging images within an organization, easy access and exchange of images between organizations has yet to be realized on a large scale.

Addressing these needs has remained a challenge despite the contemporary advancements in interoperability in other aspects of healthcare, such as Consolidated Clinical Document Architecture (C-CDA) [3] and Fast Healthcare Interoperability Resources (FHIR) [4]. This is for multiple reasons, including:

- Technical standards adoption. While there are open technical solutions for image exchange, such as Integrating the Healthcare Enterprise (IHE) Cross-Enterprise Document Sharing for Imaging (XDS-I) [5], that have gained adoption in some countries, they have not gained widespread adoption in other markets (e.g., the US market). Many of the solutions in the USA are still proprietary.
- Image type standardization [6]. Though one might immediately think of the Digital Imaging and Communications in Medicine (DICOM) [7] standard when discussing medical imaging, providers also need solutions that address images and related content in other formats, such as a JPEG image of a wound captured with a digital camera or a PDF of an EKG tracing. Two approaches have been considered, though most practical experience focuses on the first:
  - DICOM
    - DICOM-wrapping of non-native DICOM object types (e.g., JPEG, MPEG, and PDF)
  - Mixed file formats, including DICOM, JPEG, MPEG, and PDF.
    - Exchanging in the original binary file format, both DICOM and others
- Governance [8]. Successful image exchange requires more than just technology—a trust framework is needed to address several key considerations, including:
  - Which organizations will participate, and for what use cases?
  - Which individuals have access to look at images? How is that access managed?
  - What is the breadth of participants? (State? National? International?)
  - What is the remediation path to address concerns that may arise?
  - How will participants identify patients within the exchange network?
- Business incentive. Implementation of an image exchange requires a business case to justify the cost and responsibilities it implies. In the USA, the traditional fee-for-service model is gradually shifting towards a performance-based system that incentivizes outcomes and reduced cost. Novel programs, such as Accountable Care Organizations (ACOs) [9], will benefit from image exchange by reducing the cost of unnecessary duplicative testing.
- Security and Privacy. Increased flow of patient data between organizations must be balanced with the necessity of keeping that data safe and secure. While not discussed in this paper, secure exchange and privacy must be considered in an image exchange solution.

In this paper, we will look at three different standards-based image exchange implementations that have been deployed to facilitate exchange of images between provider organizations. The paper will describe how each implementation uses applicable technology and standards; the image types that are included; and the governance policies that define participation, access, and trust. Limitations of the solution or non-standard approaches to solve challenges will also be identified.

Much can be learned from successes elsewhere, and those learnings will point to recommendations of best practices to facilitate the adoption of image exchange.

## Three Standards-Based Image Exchange Implementations

### Implementation #1: Ontario, Canada

In Canada, the emphasis has been on the distribution and sharing of health records across disparate hospital and clinic organizations. In 2001, a federal government agency, Canada Health Infoway (CHI) [10], was established with the goal of achieving “one patient, one record.” The intent of this goal was to achieve seamless sharing of a patient’s health records, regardless of the location of the patient or location of the clinician requiring access to the health records.

The responsibility of sharing diagnostic imaging (DI) and reports was assigned at a provincial and regional level. This allowed each region to employ different methods appropriate for the province’s size and needs. As a result, there are varying approaches on how the exchange of DI exams is handled across Canada; however, in all cases, a key part of this mandate is the effective sharing and exchange of diagnostic imaging information using established standards and IHE profiles whenever possible.

#### Background and Requirements

A provincial government agency, eHealth Ontario, established four separate diagnostic imaging repositories (DIRs), one for each of 4 regions in the province. Each DIR contains a copy of all of the images generated in that region. This paper will provide an example of how two of these DIRs were able to leverage standards to exchange diagnostic imaging and reports between disparate hospitals and organizations that use a number of different PACS vendors. The two DIRs [11] highlighted cover the eastern and western parts of Toronto and surrounding areas, which represent approximately 8 million patients. Both of the DIRs use a hub and spoke model, in which spoke sites contribute to and retrieve from a central repository; however, the two DIRs employ different methods for exchanging images and reports. One of the DIRs (referred hereafter as DIR1) uses DICOM query/retrieve as the primary method of discovery and retrieval of outside exams. Outside exams are other exams in the diagnostic imaging repository that were not originally stored into and managed by that local PACS and its database. DIR1 went live 2007. The other DIR (referred hereafter as DIR2) leverages an XDS registry as a means of discovering outside exams and uses DICOM to retrieve them. DIR2 went live in 2011. The chart in Table 1 highlights some of the characteristics and differences between DIR1 and DIR2.

**Table 1:** Comparison of two standards-based approaches utilized for Image Exchange in Ontario (East Toronto and West Toronto). Both approaches utilize a centralized “diagnostic image repository (DIR)” to store a copy of the images generated in that Ontario region. But each DIR uses different methods to identify patients, discover images, and retrieve reports

Item	Diagnostic image repository #1 (DIR1) (uses DICOM query/retrieve) East Toronto	Diagnostic image repository #2 (DIR2) (uses XDS-I) West Toronto
Patient identification	Deterministic match based on provincial Ontario Health Card Number.	PIX lookup: probabilistic patient matching based on a scorecard of key demographics.
Image discovery	DICOM (C-Find, C-Move, etc.)	XDS registry query
Report retrieval	Report txt is available from a well formed URL. Edge device will scrape contents of URL and convert to ORU.	Reports stored in document repository in CDA format. Edge device will convert CDA to ORU or DICOM Secondary capture depending on site’s preference.
Number of contributing sites	98	29
Patient population	≈ 4.2 Million patients	≈ 4 Million patients
Number of unique PACS vendors connected	9	9
Annual exam	≈ 5 Million exams annually	≈ 3 Million exams annually

**Fig. 1:**
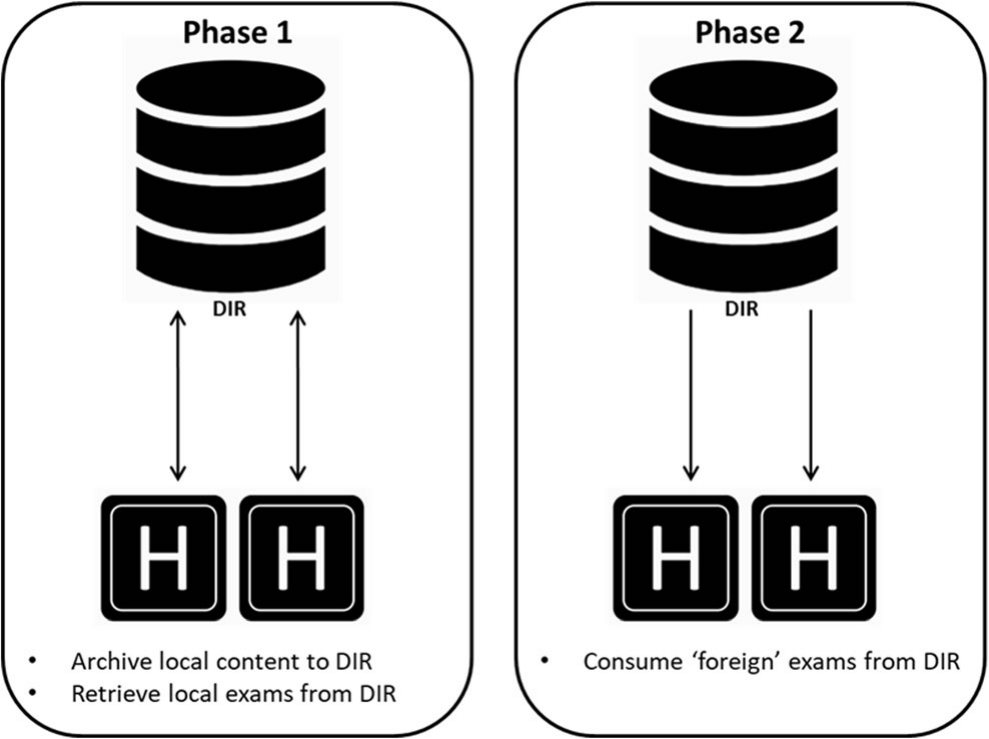
Diagnostic image repository project implementation broken into two phases

The project for both DIRs was implemented in two phases (Fig. 1). Phase 1 focused on connecting the contributing sites (many different local Picture Archive Communication Systems (PACS) in the region) to the central repository and enabling them to archive their images to the DIR. Phase 2 focused on allowing the contributing sites to consume the longitudinal record of their local patient directly into their local PACS.

Typically, a local PACS is not designed with the ability to discover and retrieve exams that do not exist within its local PACS database. As a result of this limitation, a third-party edge device (proxy-server or broker) was required to facilitate the discovery and transfer of outside imaging in both DIRs. In order for the exchange of outside imaging to be considered successful, the outside imaging exam had to meet the following requirements:

1. Will display in the local patient jacket
2. Will not re-archive back to the DIR
3. Will purge off of the local PACS after a defined period of time passed

The requirement to create unique purge rules for outside exams was created because of the risk of ingested outside exams becoming “stale” in the local PACS. This could occur if changes are made to an exam at the originating site subsequent to it being consumed by another site. As previously mentioned, a local PACS does not discriminate between a local and an outside exam; as a result, point #3 required customizations to be put into place to allow the PACS to properly manage outside exams.

#### DIR1—East Toronto

**Fig. 2:**
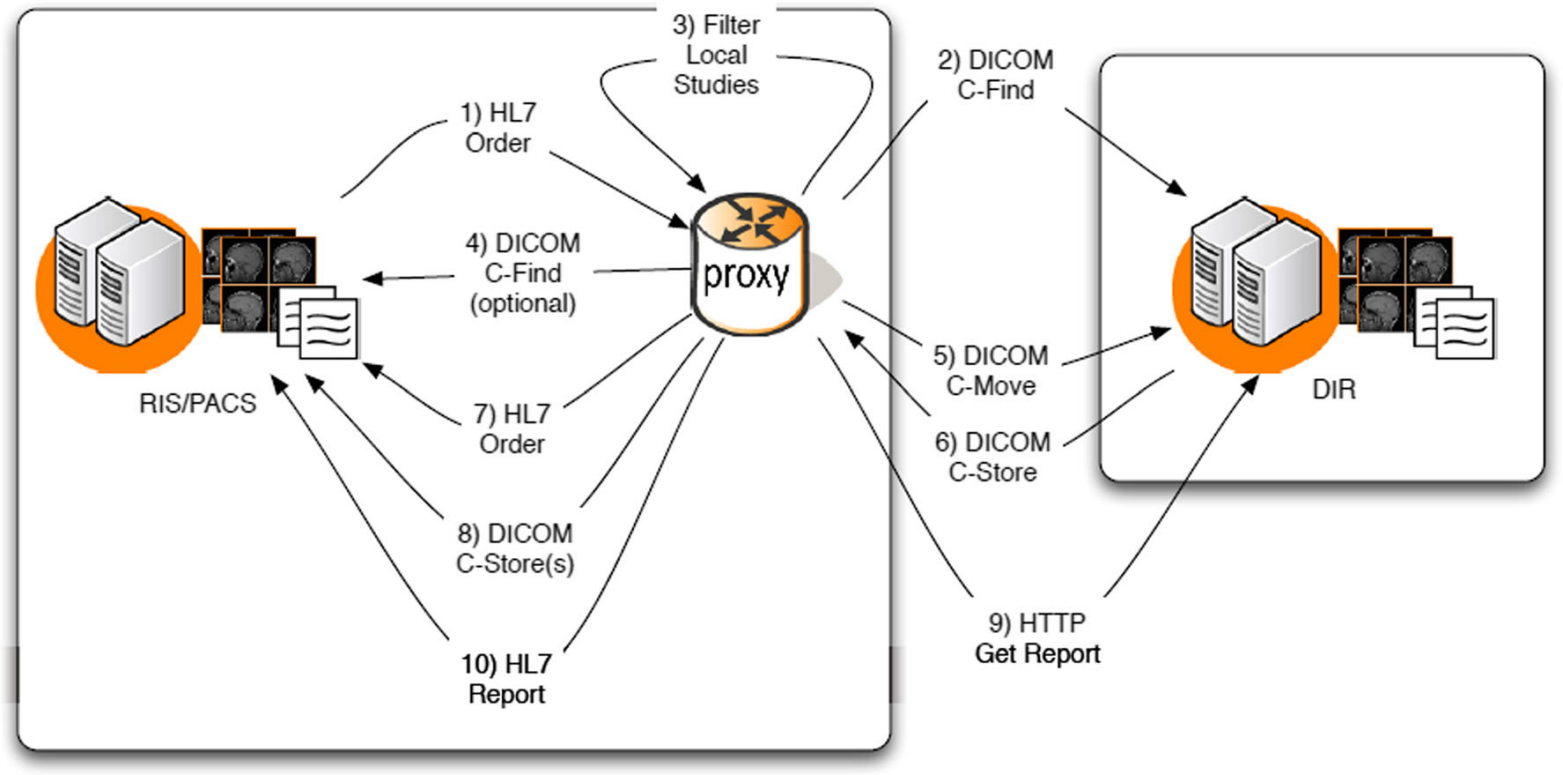
Diagnostic Image Repository1 (East Toronto): data flow for discovery and retrieval of outside exams into local PACS. The retrieval flow provides both the images and report and precedes these with order if the PACS requires it

Diagnostic imaging repository 1 (DIR1) covers the eastern part of downtown Toronto and areas to the east of Toronto, with a population of roughly 4.2 million (Fig. 2). There are currently 98 sites in that region publishing diagnostic imaging and reports, generating over 5,000,000 exams annually [12].

##### DIR1 Patient Identity

The 98 contributing sites represent 40 unique patient-identity groups. In order to avoid Patient ID collisions in DIR1, the DICOM tag “Issuer of Patient ID” (0010,0021) is used to identify the source of the Patient ID and ensure uniqueness. This allows multiple sites to use the same local Patient ID for different patients; however, a data collision will not occur because the DIR distinguishes uniqueness based on a data couplet of Patient ID and issuer of Patient ID.

DIR1 does not use an enterprise master patient index (EMPI) to match a patient’s longitudinal records. DIR1 uses the Ontario (province unique) health card number (HN) as a means of determining a patient match across multiple sites. Contributing sites are required to include the patient’s HN in the HL7 ADT and ORM messages. The DIR maps the HN to the DICOM tag, “Other Patient ID Sequence” (0010,0002), which provides a global patient identifier across all of the connected sites.

##### DIR1 Discovery and Retrieval Flow

When a health level 7 (HL7) order message (ORM) is sent from the site’s local environment to the proxy, the pre-fetch of outside imaging is triggered. The proxy will perform a DICOM C-FIND query based on the local Patient ID contained in the ORM and the Issuer_of_Patient ID derived from the sending facility in the ORM. The DIR provides C-FIND results based on patients with a matching Ontario HN, which is located in the DICOM attribute “Other Patient ID Sequence” (0010, 1002).

From the search results, the proxy disregards local exams by filtering exams with a corresponding local Issuer_of_Patient ID. A DICOM C-Move will transfer the images from the DIR to the proxy. Depending on the ingesting PACS, an HL7 order may be required. If the ingesting PACS requires an ORM, the proxy will construct and submit an HL7 ORM to the PACS.

Currently, among the ingesting sites and the present DIR solution, there is no native support for the DICOM tag “issuer of accession number sequence.” Without this value, it is difficult to identify the assigning authority that issued the accession number. To ensure that the originating site can be identified by the accession number and to avoid potential duplicate accession numbers, an alternative method was utilized that assigns a prefix for the originating site to the accession number. As a result, the ORM will contain a localized Patient ID, an accession number pre-fixed with a site identifier related to the originating site, and a procedure code that exists in the local PACS dictionary.

##### DIR1 Study Report

During the initial site integration phase of the project, none of the ingesting PACS fully supported the display of DICOM SR. The opportunity of leveraging DICOM SR is being considered in future versions of image exchange. Due to the inability of ingesting sites to view SRs, the DIR provides the reports via a well-formed HTTP URL.

The proxy is then able to “scrape” the contents of the URL and create an observation result (ORU) message. After the final image has been accepted, transformed, and forwarded to the PACS, the proxy will retrieve the associated report from the DIR’s HTTP URL.

#### DIR2—West Toronto

DIR2 covers the western part of downtown Toronto and areas to the west of Toronto, with a population of 4 million. There are currently 29 sites publishing diagnostic images and reports, resulting in over 3,000,000 exams annually [12].

##### DIR2 Patient Identity

**Fig. 3:**
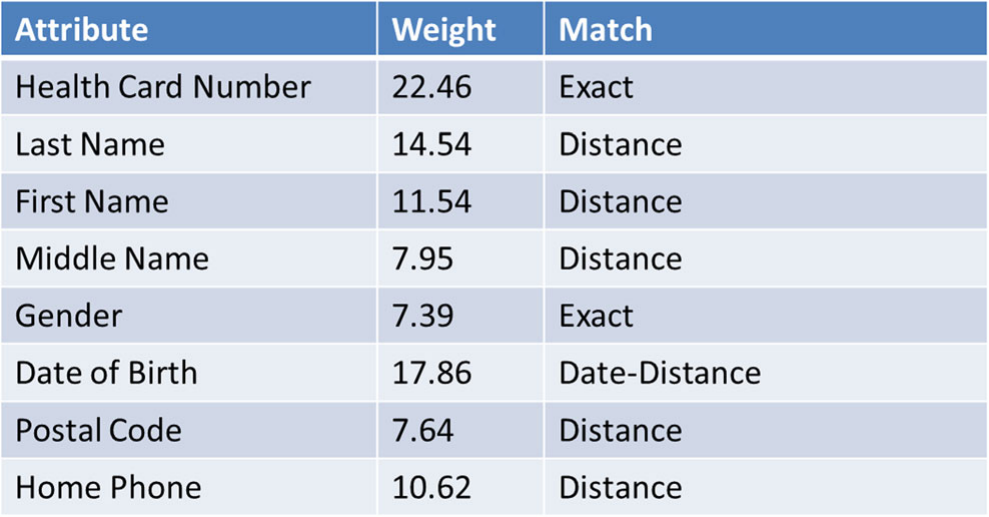
Diagnostic Image Repository2 (West Toronto): patient identity scorecard indicating the weighting values assigned for each attribute. "Distance" indicates that a calculation is made for that attribute to determine how close it is to exact. The full weighted value would be assigned if there is an exact match. Something less than the weighted value will be assigned if the match is not exact

The 29 contributing sites represent 21 unique patient-identity pools. DIR2 uses an EMPI to match a patient’s longitudinal records. Patient demographics are compared and measured against a scorecard (see Fig. 3) with the following results:

- A score greater 70 is considered a positive patient match.
- A score less than 63 is considered a different patient record.
- A score 63–70 is considered an uncertain link and requires human intervention to review and manually match or break the “uncertain link.”

##### DIR2 Discovery and Retrieval Flow

**Fig. 4:**
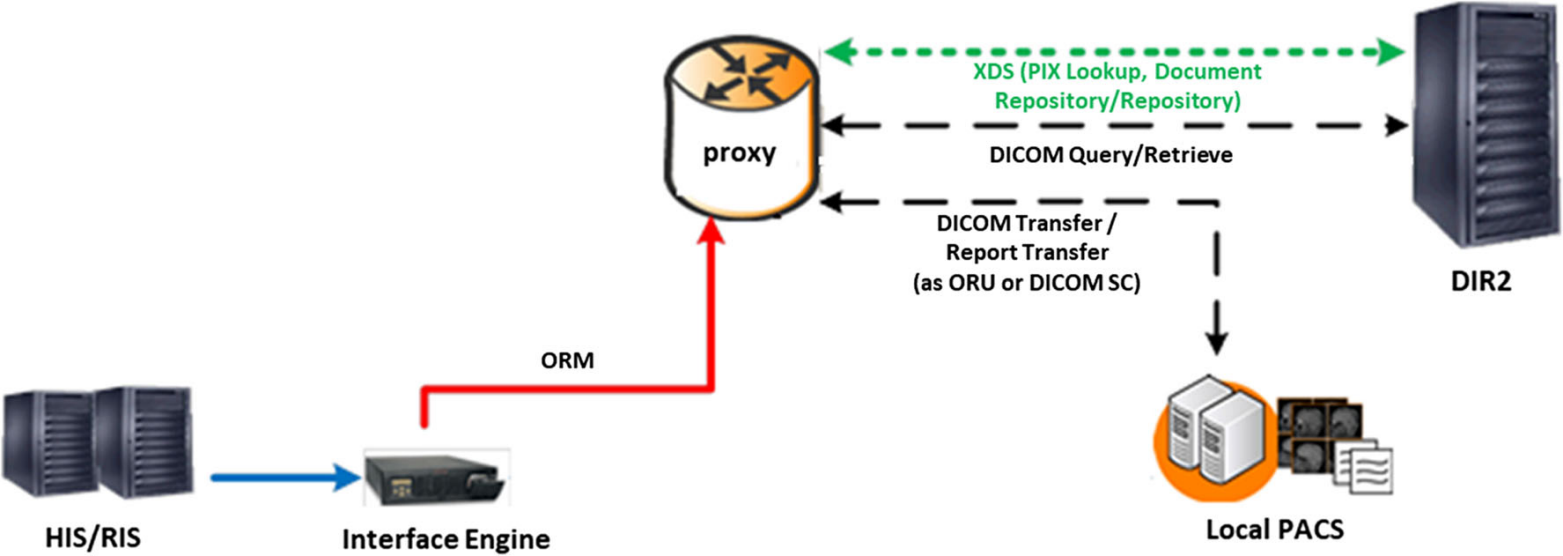
Diagnostic Image Repository2 (West Toronto): data flow for discovery and retrieval of outside exams into local PACS. The retrieval flow provides both the images and report and precedes these with order if the PACS requires it

Similar to DIR1, when a HL7 ORM message is sent from the site’s local PACS to the proxy, the pre-fetch of outside imaging is triggered (Fig. 4). The proxy uses details in the ORM to determine which pre-fetch rules that will be applied (i.e., relevancy and date range). Additionally, the demographic details in the ORM are used to authenticate the PIX/PDQ Manager node and issues a PIX query (IHE ITI-9 transaction) to determine the patient’s regional ID.

The proxy employs the patient’s regional ID to issue the appropriate Registry Stored Query (IHE ITI-18 transaction) and filters the results to include only outside imaging. The results of the outside imaging are localized to correspond with the local MRN and local terminology lexicon and filtered to identify clinically relevant outside exams based on defined pre-fetch rules. The proxy writes the metadata to the database which triggers an entry in the route queue to pre-fetch the outside imaging. If required by the ingesting local PACS, an HL7 ORM message related to the retrieved exam is created by the proxy. The proxy authenticates the XDS Document Repository node, retrieves the report from the XDS Document Repository, converts the report from a CDA format to a DICOM Secondary Capture (SC), and adds it to the route queue for data transfer to the PACS. Once the PACS is prepared to receive the outside study and the report is in the route queue, the proxy performs a DICOM C-MOVE request to the transfer the objects to the local PACS.

##### Assessment

**Fig. 5:**
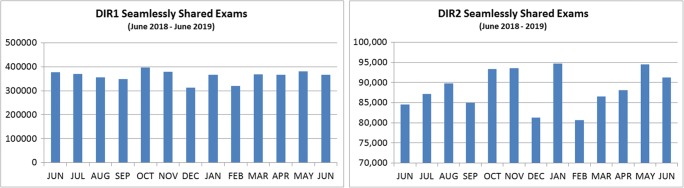
Number of exams retrieved per month over a 13-month period from DIR1 and DIR2

As a means of measuring the success of the implementation, an analysis examining the relationship between a patient’s local imaging and their longitudinal imaging history over the course of a 13-month period was conducted. The analysis looked at the period from June 1, 2018 to June 30, 2019 and measured how many foreign (outside) exams were consumed across the all the hospitals from their respective DIR. Figure 5 indicates the number of foreign (outside) exams that were exchanged from DIR1 and DIR2 respectively during that time period.

DIR1’s exam sharing volumes are consistent and steadily exceed more than 300,000 foreign exams shared across the connected sites per month. In contrast, DIR2’s exam sharing volumes are significantly lower, never exceeding 95,000 foreign exams shared in a single month. The disparity between the two DIR’s sharing patterns has a lot to do with how site’s pre-fetch rules were applied for each implementation. DIR2 applied pre-fetch rules for outside imaging based on “relevant” body part and procedure description, whereas DIR1 applied broad pre-fetch rules to build more awareness to the clinical user of the patient’s complete longitudinal imaging record.

Other benefits that have been measured [13] as a result of enabling the exchange of diagnostic imaging exams across disparate organizations include:

- Physician reliance on access to outside images and reports for patient care
- Reduction in CD imports
- Reduction in repeat imaging for patients

### Implementation #2: ELGA in Austria

#### Background, Approach, and Architecture

In 2008, the federal entity (Republic of Austria) and Austria’s nine provinces, as well as social security, committed themselves to setting up and implementing an electronic health record in Austria (ELGA [14, 15]). Although not the initial area of focus, the plan includes the ability to access a patient’s medical images along with other medical record content.

ELGA oversight provides the governance, policies, and requirements for key aspects of the architecture, as well as regulations for harmonization and privacy of the data. The ELGA Act (ELGA Law), which passed in November of 2012, provides the legal framework for the project, including the requirement for mandatory participation by healthcare providers, with citizens having the option to opt out if they desire. ELGA law describes in detail what data are allowed to be exchanged under which circumstances and for what reason and using which standards. Health data mentioned in ELGA law currently includes discharge summaries, radiology reports, laboratory reports, and nursing reports. All of these are in a well-defined structure based on Clinical Document Architecture (CDA) [15].

A private company (ELGA GmbH [16]) was formed in 2009 to coordinate and implement the initiative. ELGA GmbH can be thought of as the pacemaker for ELGA. It is responsible for coordinating the implementation and operations, integration testing, and the development of future e-Health applications. It also has responsibility for the implementation architecture and document structure.

The ELGA implementation is strictly standards-based for semantic and technical interoperability and is compliant with the applicable IHE (Integrating the Healthcare Enterprise) integration profiles. CDA® Release 2 [17] was chosen as the relevant document standard because it met the stipulated requirements. The CDA standard was developed on an XML basis in 2005 by Health Level Seven International (HL7). Existing local HIS or EHR systems can be connected to ELGA by adding an IHE connector and a CDA manager.

The ELGA architecture is implemented as a distributed, decentralized IT system with several centralized components.

The decentralized components include:

1. Health data: with the exception of images, health data is stored locally as CDA documents in decentralized XDS repositories in each Austria region (Affinity Domain).
2. Images: the original images may remain in the PACS/VNA in the Austria region where they were originally created or they may be archived in AURA (Austrian Radiology Archive) if the local radiology practice has contracted with AURA. In either case, an imaging manifest is created as a DICOM KOS for each study and stored in the XDS repository for that region. Note that for images stored in AURA, AURA acts as its own region (Affinity Domain).
3. XDS Registry: an XDS registry provides a list of documents available for each patient in that region “(Affinity Domain).
4. XCA Gateway: supports an outside query (against the local XDS registry) and retrieval (from the local XDS repository) of documents available for each patient.

The centralized components provide for:

1. Identification of the patient: The decentralized XDS data repositories (XDS Affinity Domains) are linked by means of the Centralized Master Patient Index (C-MPI) to ensure unambiguous patient identification.
2. Identification of the health service providers: The proper setup of a treatment context requires identification of the organization where the treatment is taking place and the identification of the physician or nurse involved. This is managed as a central service.
3. Access management and auditing: Manages roles and rights for ELGA consumers, provides the basis for security-oriented access to ELGA, and provides auditing services.

The first regions of Austria went live with ELGA in 2015. The last region in Austria was connected at the end of 2018. Currently, hundreds of public hospitals are connected to ELGA with their EMR, RIS, and LIS systems. For the hospitals, this was a significant effort since all reports needed to be harmonized.

A vendor delivered the infrastructure (MPI, EHR, Adaptor Set, etc.) and all of the professional services.

So now, it is time to add the capability to exchange images leveraging the existing operational XDS infrastructure as a foundation. A promising ELGA region for connecting and sharing images via ELGA is AURA (Austrian Radiology Archive). The “AURA®” platform is a vendor-managed solution which provides a cloud-based long-term imaging archive and an XDS Affinity Domain as an option for established radiologists, private hospitals, and other healthcare providers.

As mentioned earlier, Austria has nine provinces or regions. AURA acts like an additional ELGA region but focused on the archival and retrieval of image content for those customers that have contracted to use it. AURA already collects images—for the purpose of long-time preservation (which is 30 years based on Austrian law) from some private radiology practices and institutes. The intent is to make them available over ELGA mechanisms as soon as possible.

The ELGA Access Management and Auditing central service does not accommodate the exchange of images at this time. This is currently in the implementation phase along with adding support for other data types such as vaccination data, patient summaries, cross-border data exchange with other European countries, Europe-wide authentication, terminology translation, order entry for laboratory, etc.

#### Challenges and Learnings

One of the current challenges regarding the exchange of images is how to implement and manage the ELGA privacy requirements (regulated by ELGA law) and the general data protection requirements (GDPR) based on recent EU law. GDPR law requires that both the patient and the originator of the images must agree for each purpose before the images can be shared for that purpose. It also places restrictions on how the data (images) can be used after they are shared. There are ongoing legal discussions around this topic. However, the image exchange technical work is proceeding. Vienna will be the first region to connect the PACS and archiving systems of its hospitals to the ELGA network. The plan is to evaluate and summarize this experience and what was learned before expanding image sharing to other regions of the country.

Other current challenges (questions to be answered) around image management, exchange, and access include:

- When should the images be registered (DICOM KOS for the imaging manifest created) resulting in an update in the XDS registry?
- What to do if a series that has already been registered is updated?
  - Should a new KOS be created/registered or should the old one be updated?
- How to integrate the APPC (Austrian PACS Procedure Codes) in the KOS?
- How to deal with thumbnails?
- How to deal with the fact, that the patient portal in ELGA (which is delivered from another vendor) does not support images at the moment?
- How patients can withdraw their consent. ELGA has an opt-out system, rather than opt-in. This means that every patient automatically participates in ELGA—patients must explicitly withdraw their consent if they wish to opt-out

In the early stages of the project (~ 2012), one XDS Region in Austria, which contains 13 hospitals, piloted image exchange with XDS-I using their local repositories. It worked reasonably well; however, these hospitals discontinued the pilot as they had to prioritize their limited resources to comply with the ELGA law.

#### Opportunities

On the list of opportunities, IHE-WIA (Web-based Image Access) [18] is one of the profiles being considered for a pilot project. WIA would allow retrieval of the images using DICOM Web Services. WIA offers many benefits such as:

- Provides a mechanism to query/retrieve DICOM imaging objects using DICOM RESTful web services enabling ease of image access on non-traditional DICOM devices (e.g., mobile devices)
- Can leverage and utilize the existing XDS-I infrastructure
- Can access related imaging documents by integrating with IHE MHD (mobile access to health documents)
- Easy to work across firewalls using HTTP(S) compared to traditional DICOM (DIMSE)
- Easy to support authentication and encryption using HTTPS compared to DICOM
- Not dependent on a secure network (VPN) between endpoints

### Implementation #3: USA—RSNA Image Share Network

The RSNA Image Share Network was developed to provide a model and operating pilot for expanding patient access to images and reports. The project was launched in 2009 under a contract funded by the National Institute of Biomedical Image and Bioengineering (NIBIB). The contract award specified the project goal of enabling standards-based *patient-focused* image sharing, in line with other patient empowerment initiatives driven by the National Institutes of Health. The working thesis was that empowering the patient—the actor with the strongest motivation to gain and share access to his or her records—might address some of the barriers mentioned in the introduction.

#### Approach

RSNA worked with a consortium of research sites to build and deploy a software component (the Edge Server) that connects local radiology systems (RIS/PACS) to an IHE XDS-I based network. The software tools developed under the program are freely available under an open source license on a public code repository [19]. The network infrastructure, including a data registry/repository (the Image Clearinghouse) and image-enabled personal health record (PHR) accounts, was provided by vendor subcontractors; ultimately, two vendors—lifeIMAGE and Ambra Health—supported the majority of the 35,000 patients enrolled. NIBIB contract funds were used to develop the Edge Server software, to offset the costs of implementation and maintenance for participating sites, and, for part of the project period, to support part-time research coordinators to help enroll patients. Patients were not charged for this service.

**Fig. 6:**
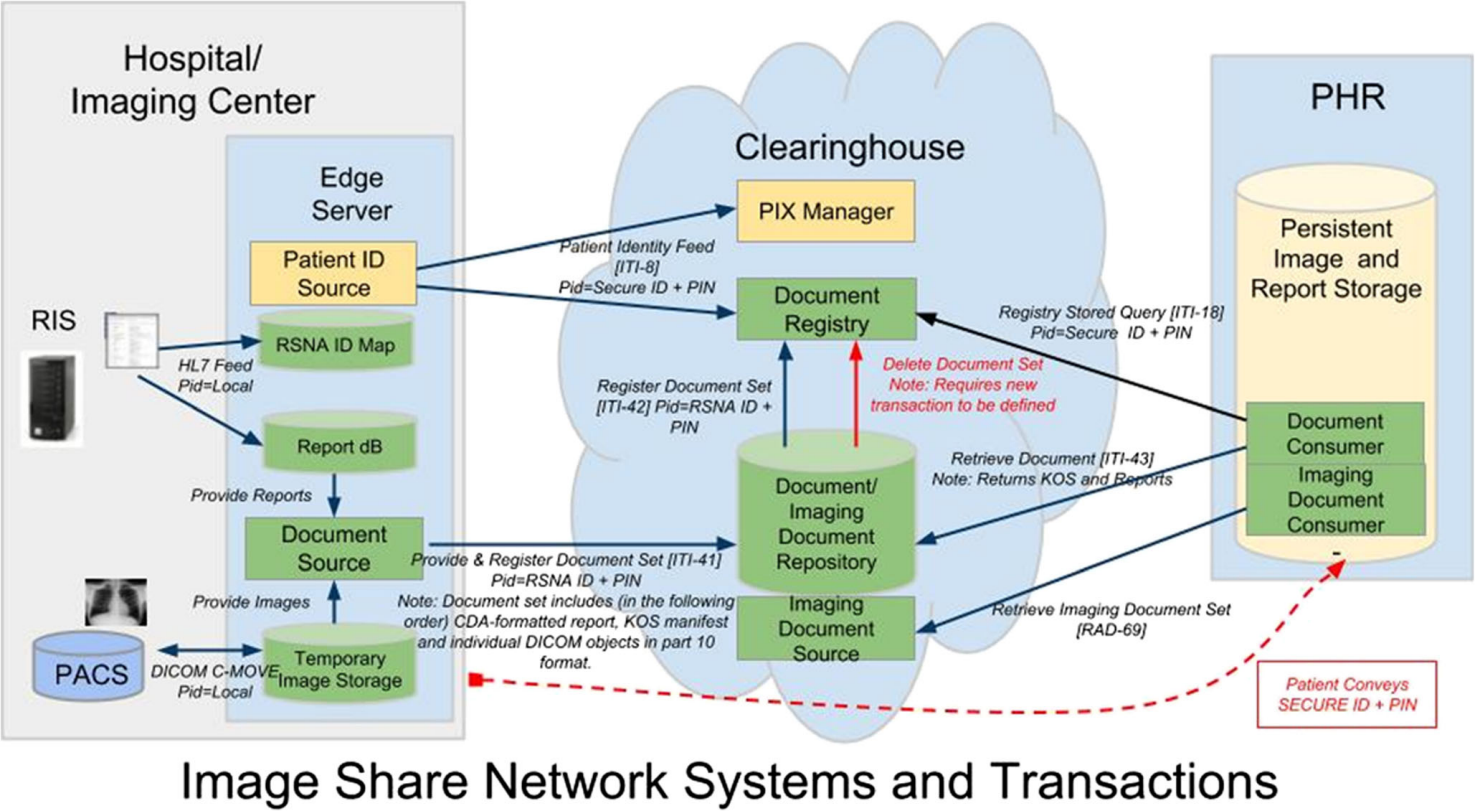
Architecture of the RSNA Image Share. In this implementation, the patient, rather than another Hospital or Imaging Center, is the active agent of exchange

The architecture of the Image Share Network (Fig. 6) is based on the IHE XDS-I profile, with modifications to support the patient-focused model. IHE XDS is designed mainly to support site-to-site sharing in health information exchanges, where it is paired with IHE profiles (notably, Patient Identity Cross Reference and Patient Demographic Query—PIX/PDQ) that enable subject discovery across disparate patient identity domains. Image Share replaced the subject query model found in such settings by instead making the patient the active agent of exchange. At the time of the patient visit (or upon subsequent request), patients were provided with a security token (a nine-digit code) and instructions they could use to establish a personal health record (PHR) account and retrieve their records into that account. With this approach, the patient exercised full control over their imaging exams without returning to the originating radiology site. This included access to the report, viewing the images in a browser, the option to download the full DICOM package, and the ability to authorize and provide access for subsequent care providers to view or download their images via an email containing appropriate links. Such authorization could also be removed by the patient.

#### Outcomes

Surveys conducted during the project showed a strong patient preference for using an online PHR over physical media (CDs and DVDs). However, the project’s success in establishing a model for image exchange is harder to judge. Over the course of the project, which ended in March 2018, the RSNA Image Share Network was used to connect 20 radiology sites and enabled over 35,000 patients to gain access to their images and reports in personal online accounts. While significant, these results were well below targeted goals. A variety of sociologic and regulatory issues proved to be impediments to more widespread adoption. These include the effort needed for outreach, communication and recruiting patient participants, security and privacy concerns, and the governance overhead of negotiating business associates agreements with each participating site. Security reviews at sites interested in deploying this solution often took a year or longer. In addition, implementation and configuration of the Edge Server, while only moderately technically complex, were subject to competing demands and priorities for on-site IT resources: the Image Share project coincided with the deployment of new Electronic Health Record (EHR) systems spurred by the Meaningful Use program, which heavily taxed those resources.

One of the RSNA Image Share project goals was to demonstrate a working solution to foster and encourage the use of standards for image exchange. The RSNA Image Share investigators recognized that utilizing a Patient Health Record model is one of several possible approaches to applying standards to implement an image-exchange solution. To continue encouraging the use of standards for image exchange, RSNA partnered with the Sequoia Project, an organization dedicated to promoting health information exchange, to conduct the RSNA Image Share Validation Testing Program. Vendors whose image share products demonstrate conformance with this set of standards receive the Image Share Validation seal. To date, nine vendors have successfully completed validation testing under the program. A new round of testing was initiated in the Summer of 2018.

The RSNA Image Share has extended its relationship with the Carequality arm of the Sequoia Project. Carequality can be thought of as a network of networks, with a tightly constrained data use agreement directed at the exchange of healthcare information. The rules of this exchange network are specified in an implementation guide (IG). Together, current work is being performed to extend the IG to include the technical specifications which would allow image exchange vendors who own client-based image exchange networks, and other standards compliant image exchange networks, to exchange exams with one another.

#### Expanding RSNA Image Share to Support Image Exchange for Research Using Emerging Standards

**Fig. 7:**
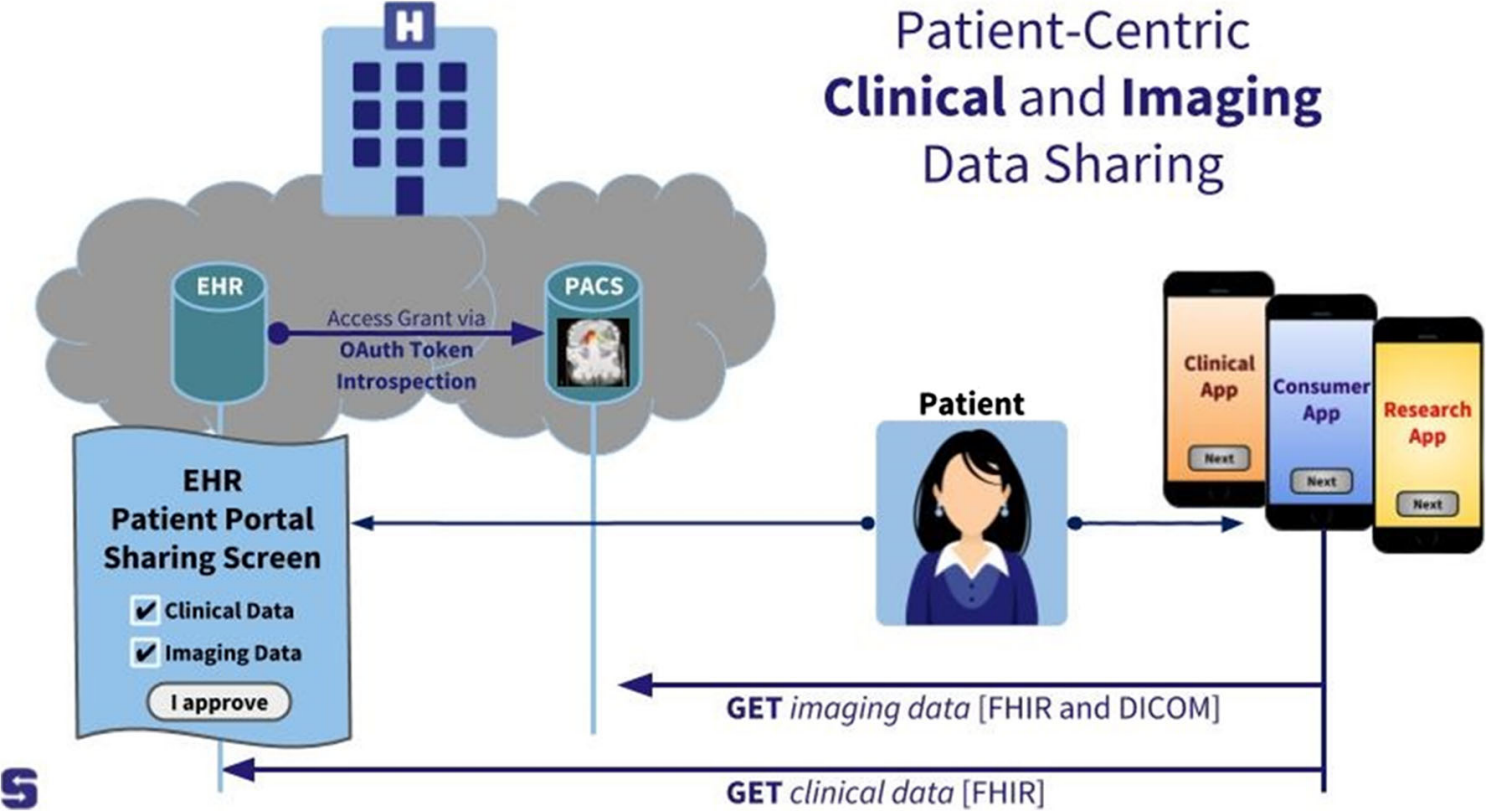
RSNA Image Share working reference implementation to enable Sync 4 Science to share images and imaging-related data using standards-based RESTful web services

In 2017, as part of a 1-year extension of the project, NIBIB directed RSNA to conduct work to enable image sharing in Sync for Science (S4S)—All of Us, one of the programs associated with the NIH’s Precision Medicine Initiative. The goal of the S4S program is to make it easier for patients to share their medical records for research. The Image Share project team developed a working reference implementation that connects current radiology systems to an application programming interface that enables response to queries for information from authorized research applications, thus adding medical images and reports to the body of records that can be shared through S4S (Fig. 7).

The standards architecture of S4S uses the SMART on FHIR [20] application programming interface (API), a set of open specifications to enable applications to connect to EHR systems. The Image Share/S4S reference model combines HL7 FHIR queries and DICOM Web responses. Both of these emerging standards are based on the RESTful interfaces used widely to support web services and web apps. The RSNA Image Share developers intend to bring this forward to the IHE Radiology domain for incorporation into the XDS-I family of profiles.

S4S has recently proposed expanding the focus of its work beyond the research use case to incorporate patient-centric sharing. While still early in its development, this architecture offers the promise of simplified deployment, closer integration between radiology systems and EHRs, and extending image access to consumer-facing applications and mobile devices.

## Conclusion and Recommended Advocacy

What can we learn from these 3 efforts towards setting up a successful, and practical, standards-based image exchange that enables a large group of providers and patients to discover and utilize a patient’s comprehensive medical imaging record?

1. Current standards are adequate to enable image exchange, and the standards continue to evolve. The availability of adequate standards, however, is not alone sufficient to establish image exchange as a regular practice, as the three examples described in this paper demonstrate. Additional considerations must be taken beyond technological standards.
2. Having an engaged national or regional governing body tasked with enabling Health Information Exchange, including image exchange, across the nation or region, goes a long way toward successful implementation. Several significant factors for success can be provided by the governing body:
  - Specification of the standards that must be used—so everybody does it the same—is critical to enabling interoperability.
  - Creating a nation-wide, or region-wide, program that encourages participation can address a number of the key *governance* factors such as:
    Providing a single shared *trust framework* for all participants that mandates and protects availability of the data and access to it—so that individual legal agreements do not need to be negotiated between each pair of exchange partners.
    Defining what data is to be exchanged and in what standard formats it will be encoded.
    Defining the breadth of participants (international/national/regional/state/organizational/individual)
    Specifying how access is managed, secured, and audited.
    Creating a national or regional patient identifier, or standing up a patient identifier cross-referencing service, to aid in discovering, associating, and accessing the right images for the right patient.
  - To make image exchange work, core pieces of infrastructure need to be in place, and there is a cost for implementing and maintaining that infrastructure. The governing body can provide or incentivize the provision of that key infrastructure. In the absence of such a governing body, each image exchange initiative must stand up its own infrastructure, define methods for accessing the data, and create a business case that justifies the cost of doing so.

d. Both the Canadian and Austrian implementations include the governance and sustainable core infrastructure that provide most of the “factors for success” described in point 2 above. The RSNA image share successfully created a small ecosystem of patients and demonstrated that the standards can work for patients to access their images, with reports, in a Personal Health Record. To expand that model into a sustainable and effective nation-wide image exchange is still waiting for the necessary governance and funding to supply some of the key “factors for success” described above. The RSNA Image Share is currently working with Carequality to establish a governance model in the USA to enable national image exchange in the HIE model alongside the PHR model.

3. Image exchange needs to be integrated with the broader patient record in a Health Information Exchange. Images, and other multimedia content, are a valuable part of the medical record and important for providing continuity of care across medical providers. For reasons that have to do with the nature of imaging records, which are relatively large and variegated multimedia data files that require greater expense than many other medical records to store and transmit and specialized tools to manage and display, image exchange seems to be the harder part of health information exchange. Even some countries with well-established nationwide health information exchange programs have struggled to implement comprehensive image sharing.
  In the USA, while the Office of the National Coordinator (ONC) has redoubled its effort to provide the necessary governance and regulatory incentives to encourage health information exchange, it has not yet proposed including medical images in the core data set that care providers are required to exchange. The lack of national or regional governance for image exchange has meant that image-sharing vendors have had little incentive to connect their disparate user networks, allowing them to provide competitive solutions that do not necessarily follow standards that would enable interoperability. Without governance that defines a common model for health information exchange, including exchange of medical images, access to patient imaging records will not be convenient or well correlated with other related information in the medical record.
